# A Manual of Hygiene and Sanitation

**Published:** 1901-03

**Authors:** 


A Manual of Hygiene and Sanitation. By Seneca Egbert, A. M., M. D., Professor of Hygiene in the Medico-Chirurgical College of Philadelphia. New (2d) and revised edition. In one 12mo volume of 427 pages, with 77 engravings. Philadelphia and New York: Lea Brothers & Co. 1900. [Price, cloth, $2.25 net.]

The appearance of a second edition of Egbert's work on Hygiene and sanitation proves the value of the book and the widespread interest taken by the profession on this subject. So plain and untechnical is the writer's style that the lay reader, as well as student and practitioner, can find a horde of vitally interesting facts between the two covers. Nothing is more vital at the present time than the study of pure water, school sanitation, the sewage problem, disinfection and quarantine, and these are the subjects which the author discusses so thoroughly and entertainingly. In this edition the author has added a chapter on military hygiene, quoting at some length the admirable paper of Prof. Victor C. Vaughan on State Medicine, before the last meeting of the American Medical Association. The statement is made that "every regiment in the United States service in 1898 developed typhoid fever." The advisability of adding a chapter on this subject is therefore very apparent.

In every detail the work will be found a clear, comprehensive and authoritative exposition of its subject. W. C. K.

